# Glutamate Receptor Interacting Protein 1 Mediates Platelet Adhesion and Thrombus Formation

**DOI:** 10.1371/journal.pone.0160638

**Published:** 2016-09-15

**Authors:** Kristina L. Modjeski, Sara K. Ture, David J. Field, Scott J. Cameron, Craig N. Morrell

**Affiliations:** 1 Aab Cardiovascular Research Institute, University of Rochester Medical Center, Rochester, NY, United States of America; 2 Department of Pharmacology and Physiology, University of Rochester Medical Center, Rochester, NY, United States of America; University of Kentucky, UNITED STATES

## Abstract

Thrombosis-associated pathologies, such as myocardial infarction and stroke, are major causes of morbidity and mortality worldwide. Because platelets are necessary for hemostasis and thrombosis, platelet directed therapies must balance inhibiting platelet function with bleeding risk. Glutamate receptor interacting protein 1 (GRIP1) is a large scaffolding protein that localizes and organizes interacting proteins in other cells, such as neurons. We have investigated the role of GRIP1 in platelet function to determine its role as a molecular scaffold in thrombus formation. Platelet-specific GRIP1^-/-^ mice were used to determine the role of GRIP1 in platelets. GRIP1^-/-^ mice had normal platelet counts, but a prolonged bleeding time and delayed thrombus formation in a FeCl_3_-induced vessel injury model. *In vitro* stimulation of WT and GRIP1^-/-^ platelets with multiple agonists showed no difference in platelet activation. However, *in vivo* platelet rolling velocity after endothelial stimulation was significantly greater in GRIP1^-/-^ platelets compared to WT platelets, indicating a potential platelet adhesion defect. Mass spectrometry analysis of GRIP1 platelet immunoprecipitation revealed enrichment of GRIP1 binding to GPIb-IX complex proteins. Western blots confirmed the mass spectrometry findings that GRIP1 interacts with GPIbα, GPIbβ, and 14-3-3. Additionally, in resting GRIP1^-/-^ platelets, GPIbα and 14-3-3 have increased interaction compared to WT platelets. GRIP1 interactions with the GPIb-IX binding complex are necessary for normal platelet adhesion to a stimulated endothelium.

## Introduction

Platelets contain many molecules and proteins most identified with neurons such as N-ethylmaleimide factor [1,2], serotonin [3,4], glutamate, and glutamate receptors [5–8]. Our previous studies demonstrated that platelet glutamate receptor signaling increases platelet activation and thrombus formation [5–6]. Glutamate receptors are trafficked and assembled into functional complexes by multiple accessory proteins including glutamate receptor interacting protein 1 (GRIP1) [9]. GRIP1 binds to and regulates the surface expression of the AMPA receptor GluR2 subunit [10,11]. GRIP1 is a multi-PDZ domain scaffolding protein that forms homodimers that can bind and simultaneously organize and assemble protein complexes via PDZ (Post-synaptic density 95, discs large, and zonula occludens 1) domains [11]. Complete GRIP1 knockout (GRIP1^-/-^) mice have multi-organ developmental abnormalities and die due to hemorrhage and hypovolemia in the embryonic or early post-natal period [12]. Immunofluorescence of serous and hemorrhagic blisters for PECAM-1 (platelet and endothelial cell adhesion molecule-1) in GRIP1 null mice were negative, suggesting that the blisters did not arise from an endothelial defect [12]. Similar multi-organ defects have been reported in humans with GRIP1 mutations consistent with Fraser syndrome, a syndrome found with deletion of specific GRIP1 interacting proteins including Frem1 (Fras1 related extracellular matrix 1) and Fras1 (Fraser syndrome 1) [13–15]. Reported point mutations in GRIP1 result in a gain-of-function phenotype and are associated with autism, while neuronal-specific loss-of-function mice have prosocial behavior [16]. Other GRIP1 mutations have been implicated in schizophrenia [17]. Despite the multi-organ defects and hemorrhage in complete GRIP1^-/-^ mice, GRIP1 expression and function has not been studied in most other cell types, including platelets.

GRIP1 and 14-3-3 immunoprecipitation studies in HEK293 cells [18] and neurons [19] have suggested GRIP1 binds to 14-3-3. 14-3-3 is a member of the platelet GPIb-IX adhesion complex [20] and 14-3-3 binds to GRIP1 through a linker region which does not contain a PDZ domain [19]. Platelet adhesion to a damaged or activated endothelium is the first step in stable thrombus formation necessary for effective hemostasis. Platelet adhesion is mediated by GPIb-IX complex binding to von Willebrand factor (vWF) released from stimulated or damaged endothelial cells. While 14-3-3 has been shown to bind both GPIbα and GPIbβ and interact with the actin cytoskeleton [20], its mechanistic role in platelets remains unclear.

In this study, we report that GRIP1 binds to the GPIb-IX complex and that deletion of GRIP1 from platelets causes altered platelet rolling behavior and decreased thrombosis.

## Materials and Methods

### Mouse Colony Generation

Platelet factor 4 (PF4) Cre recombinase mice on a C57BL/6J background were purchased from Jackson Labs (#008535) and bred with GRIP1^fl/fl^ mice provided by Dr. Richard Huganir from The Johns Hopkins University. Male PF4-Cre^+^ GRIP1^fl/fl^ mice and WT littermates (PF4-Cre^-^GRIP1^-/-^, PF4-Cre^+^GRIP1^+/+^, PF4-Cre^-^GRIP1^+/+^) were used in all studies. Mice of 3–5 weeks of age were used in bleeding time and thrombosis studies and mice from 5–16 weeks of age were used for all other experiments.

### Mouse Tail Bleeding

Mice were anesthetized with ketamine/xylazine (IP at 80 and 12 mg/kg respectively) and 3 mm of the distal tip of the tail amputated with a #10 scalpel blade. Timing began and the tails were submerged in 37°C PBS until visual cessation of bleeding for at least 30 seconds without a re-bleed or until 15 minutes total time elapsed.

### Mouse Mesenteric Artery Thrombosis

Mice were anesthetized with ketamine/xylazine (IM at 80 and 12 mg/kg respectively) and injected with anti-GPIbβ (#X488, Emfret Analytics) *in vivo* platelet labeling antibody. Using a midline incision, the mesenteric vasculature was exposed for live imaging and kept on a 37°C stage warmer. Freshly prepared 15% FeCl_3_ (#157740, Sigma) was applied to the exposed vessel for 45 seconds using Whatman paper. Vessels were imaged for 20 minutes on an inverted fluorescence microscope (Eclipse Ti, Nikon) at 30 frames per second using an electron-multiplying CCD video camera (512SC, Quantem). Surface area of thrombi and total vessel surface area were measured at 10, 15, and 20 minutes after starting the video using NIS Elements software (Nikon). Images were obtained from videos.

### Platelet Preparation

Retro-orbital blood from anesthetized WT and GRIP1^-/-^ mice was collected into heparinized Tyrodes buffer (134 mM NaCl; 2.9 mM KCl; 12 mM NaHCO_3_; 0.34 mM Na_2_HPO_4_; 20 mM HEPES, pH 7.0; 5 mM glucose; and 0.35% bovine serum albumin). Washed platelets were prepared as described [5]. Expired human platelets obtained from the blood bank at the University of Rochester Medical Center were pelleted for lysates using PGI_2_ (#18220, Cayman Chemical).

### Platelet Rolling and Adhesion

For in vivo rolling experiments, washed platelets were labeled with 10 μM calcein green-AM (#C34852, Thermo Fisher Scientific) for 10 minutes, centrifuged for 5 min. at 2600 rpm at 25°C with PGI_2_, and resuspended in Tyrodes. GPIbα blocking antibody (#M040-0, Emfret Analytics) was added to the platelets in specified experiments. Platelets were injected retro-orbitally and calcium ionophore (A23186, #C5722, Sigma) was added to the vessel surface 2 min. after beginning video recording. Video commenced at 10 minutes. Platelet velocity was determined using NIS-Elements software (Nikon). Images were obtained from videos.

### In Vitro Platelet Activation

For flow cytometry activation experiments, washed platelets were stimulated with thrombin (#13188, Cayman Chemicals), 2-meADP (#1624, Tocris), and convulxin (#sc-202554, Santa Cruz) for 10 min. and then CD62P antibody (#553774, BD Biosciences) and the activated GPIIb/IIIa antibody JON/A (#M023-2, Emfret) were added. Washed platelets were activated with 0.5 μg/mL botrocetin (#V5625, Sigma) and 10 μg/mL human von Willebrand factor (#HCVWF-0190, Haematologic Technologies Inc.). For platelet aggregation, whole mouse blood was centrifuged for 15 minutes at 1000 rpm, platelet rich plasma (PRP) was incubated in a 1:100 dilution of CD9-APC or CD9-PE (Abcam) for 15 minutes after samples were washed and resuspended in plasma to a concentration of 50 × 10^6^ platelets/mL. Labeled platelets were mixed 1:1 and agonist-stimulated at 37°C while shaking similar to published by others [21].

### Flow Cytometry

Median fluorescence intensity was measured on a BD Accuri C6 flow cytometer and analyzed by FlowJo software (TreeStar Inc.).

### Immunoprecipitation and Western blots

Platelet lysates were prepared using 1X NP-40 lysis buffer added to washed platelets. GRIP1 (#AB5547, Millipore), 14-3-3 (#sc-629, Santa Cruz), GPIbα (#M040-0, Emfret Analytics), or GPIbβ (#M050-0, Emfret Analytics) antibodies were added to WT and GRIP1^-/-^ platelet lysates and incubated overnight at 4°C. Protein A/G beads (#sc-2003, Santa Cruz) were added for a minimum of 2 hours. Bead-antibody complexes were washed once with PBS and Laemmli running buffer was added. Tris-Glycine gels (4–15%, Bio-Rad) were used for Western blots with nitrocellulose membrane transfer. All primary antibodies (GRIP1 (#611318, BD), 14-3-3 (#sc-629, Santa Cruz), 14-3-3ζ (#sc-1019), GPIbα (#M040-0, Emfret Analytics), or GPIbβ (#M050-0, Emfret Analytics) were incubated overnight at 4°C. Infrared fluorescent secondary antibodies (anti-mouse Alexa Fluor 750, #A-21109, Thermo Fisher Scientific and anti-rabbit Alexa Fluor 680, #A-21037, Thermo Fisher Scientific) were added for detection on Licor (Licor).

### Mass Spectrometry

GRIP1 was immunoprecipitated (#sc-29834, Santa Cruz) from WT and GRIP1^-/-^ platelets and the immunoprecipitate was given to the Proteomics Core at the University of Rochester for band excision, trypsin digestion, and LTQ LC/MS Linear Ion Trap mass spectrometer analysis. Protein identities were assigned to GO categories by Panther (Protein Analysis Through Evolutionary Relationships) [22].

### Animal Care

All experiments involving animal procedures were approved by the University of Rochester Institutional Animal Care and Use Committee.

### Statistics

Mean and S.E.M. was calculated for all graphs. * 0.05 > p, ** 0.01 > p, *** 0.001 by two-tailed students T test for all analysis unless using Kaplan-Meier survival curves as indicated. Graphs were created with Prism Graphpad 4.0 (Graphpad).

## Results

### GRIP1 is expressed in platelets

**Fig 1:**
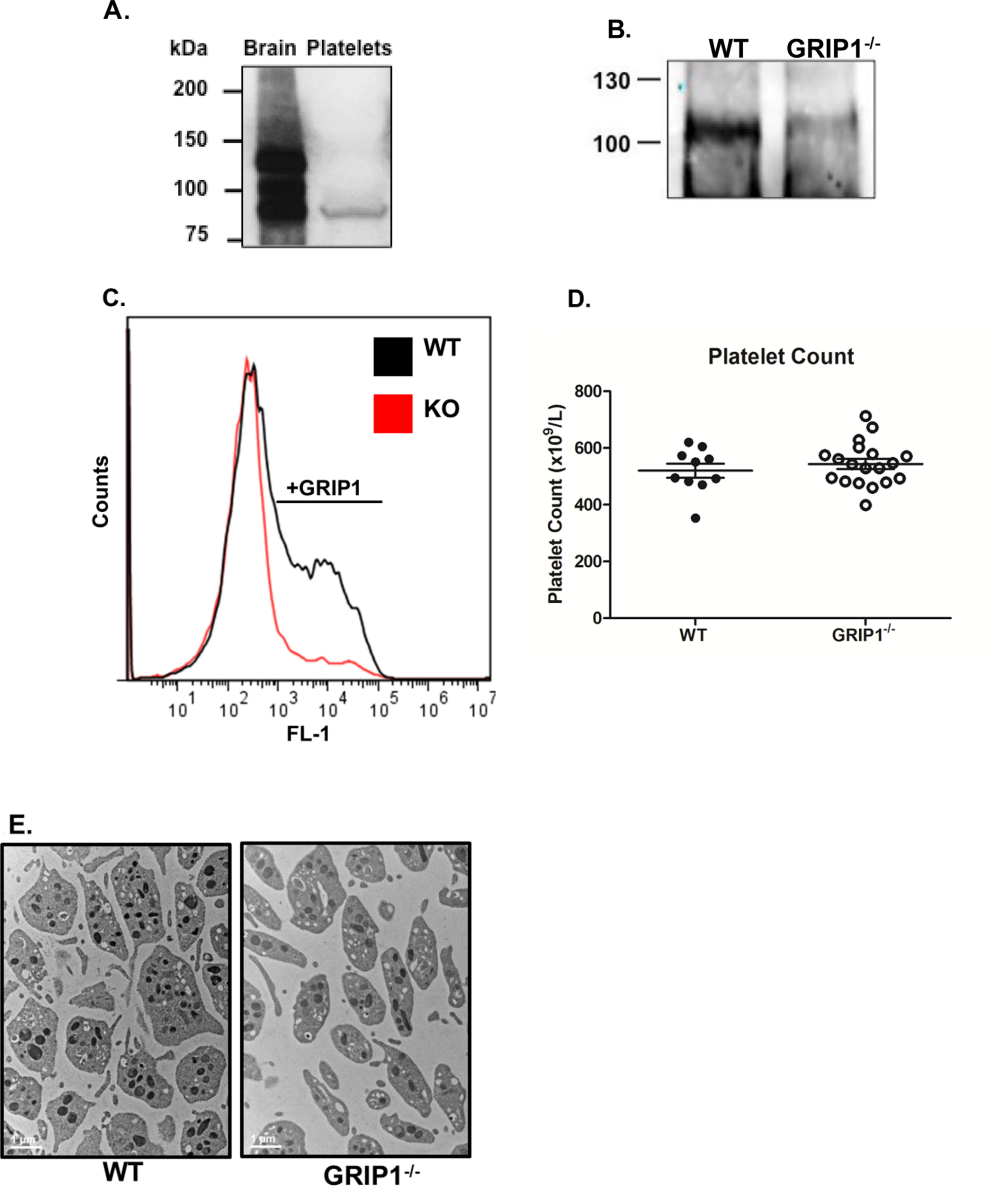
Platelets express GRIP1. A) Human platelets express GRIP1 (representative immunoblot with mouse brain as positive control). B) PF4-Cre^+^ GRIP1^fl/fl^ mice have reduced platelet GRIP1 expression. Immunoprecipitation of WT and GRIP1^-/-^ mouse platelets using anti-GRIP1 antibody and immunoblot for GRIP1. C) Representative histogram of GRIP1 expression in WT and GRIP1^-/-^ mouse platelets by intracellular flow cytometry. D) WT and GRIP1^-/-^ mouse platelets have similar platelet counts. (± S.E.M., NS by students T-test). E) WT and GRIP1^-/-^ platelets have similar morphology (representative electron microscopy of resting platelets).

GRIP1 is a large multi-PDZ domain protein that has multiple isoforms [11, 23, 24]. Using mouse brain and human platelet lysates we found that human platelets express GRIP1 protein that is consistent in size with the lowest molecular weight isoform (Fig 1A). Complete GRIP1^-/-^ mice are embryonic lethal or have severe developmental defects in multiple organ systems at birth [12]. To determine a functional role for GRIP1 in platelets, GRIP1^flox^ mice were crossed with PF4 (platelet factor 4) Cre^+^ mice to generate platelet-specific GRIP1^-/-^ mice. Platelets from PF4-Cre^+^GRIP1^flox/flox^ mice (platelet GRIP1^-/-^) have reduced GRIP1 protein compared to platelets from wild-type (WT) mice (Fig 1B). This was confirmed by intracellular flow cytometry for GRIP1 that also demonstrated reduced expression in GRIP1^-/-^ platelets (Fig 1C). The GRIP1^-/-^ mice retain some GRIP1 protein expression, but represent about a 75% reduction based on the intracellular flow cytometry. This may indicate that GRIP1 is an early expressed protein in megakaryocyte differentiation, prior to PF4 expression. Platelet GRIP1^-/-^ and WT mice had similar complete blood counts (not shown), including similar platelet counts (Fig 1D). Platelet morphology was also unchanged in platelet GRIP1^-/-^ mice as assessed by electron microscopy (Fig 1E).

### GRIP1^-/-^ mice have thrombosis deficits

**Fig 2:**
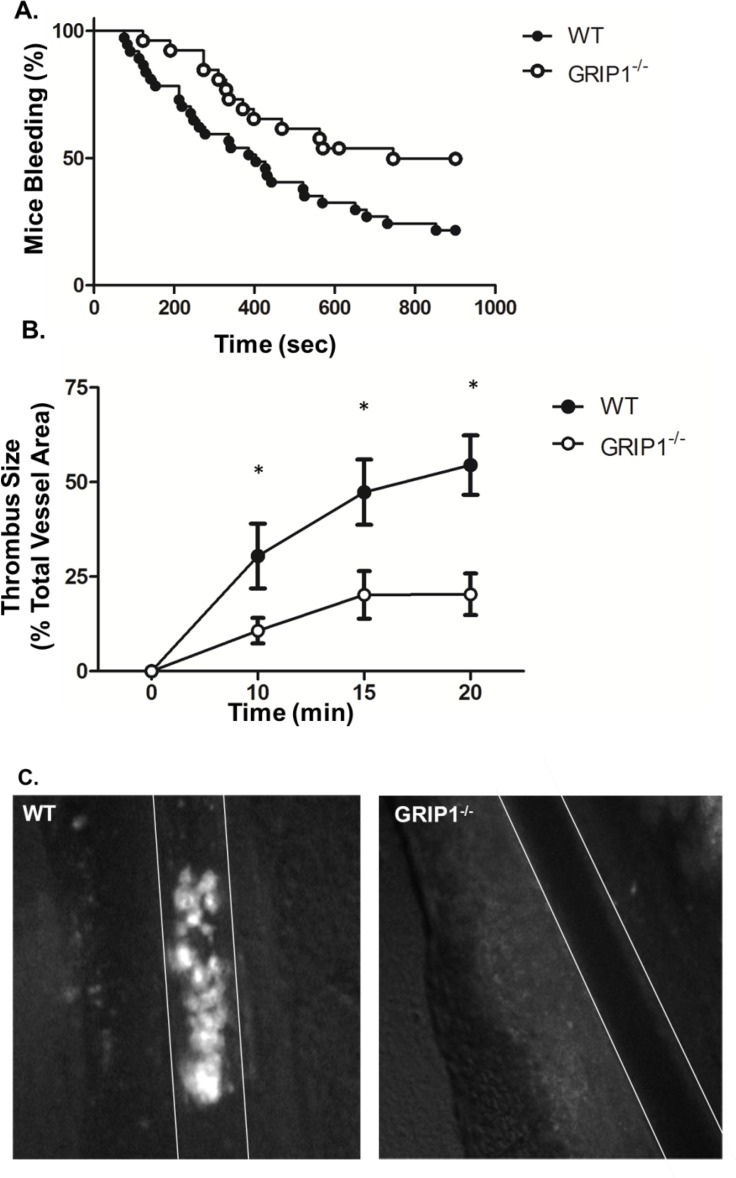
GRIP1^-/-^ mice have hemostasis and thrombosis defects. A) WT and platelet GRIP1^-/-^ mouse bleeding time. Percentage of patent vessels after 3 mm tail tip amputation (N = 25–37; P < 0.05 for Kaplan-Meier curve). B) Platelet GRIP1^-/-^ mice have reduced thrombus size. FeCl_3_-induced mesenteric artery thrombosis. Fluorescent thrombus burden expressed as percentage of vessel area (N = 11–17; ± S.E.M., *P < 0.05 by students T-test). C) Representative image of thrombus formation in WT and platelet GRIP1^-/-^ mice 10 min. after injury (dashed lines represent vessel edges, magnification 20X).

To determine whether platelet GRIP1^-/-^ mice have a change in in vivo platelet function we performed a tail bleeding time assay. The distal tail of WT and platelet GRIP1^-/-^ mice was amputated and placed in saline. The time until visual cessation of bleeding was recorded. There was a significant increase in the number of platelet GRIP1^-/-^ mice still bleeding 15 minutes after tail tip removal compared to WT mice (Fig 2A). To examine platelet thrombosis we utilized a ferric chloride vessel injury model. Ferric chloride was applied to mesenteric arterioles of WT and platelet GRIP1^-/-^ mice and platelet accumulation and thrombus formation was recorded. Platelet GRIP1^-/-^ mice had significantly decreased thrombus size after vessel injury (Fig 2B and 2C). Together these data demonstrate that mice lacking GRIP1 have prolonged bleeding and delayed time to thrombus formation.

### GRIP1^-/-^ platelets have normal agonist activation but deficient adhesion in vivo

**Fig 3:**
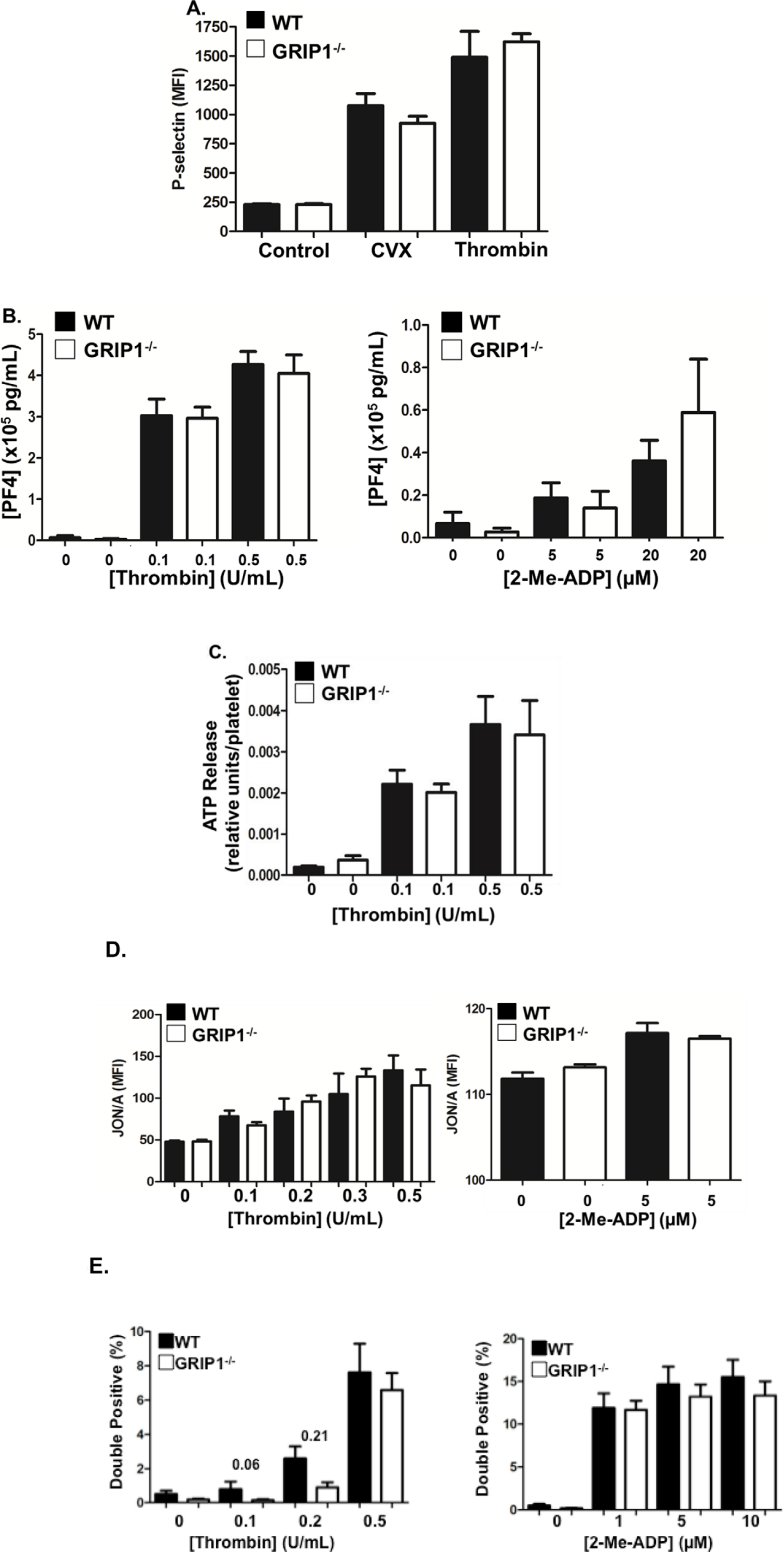
*In vitro* platelet activation is normal in GRIP1^-/-^ platelets. A) WT and GRIP1^-/-^ platelets have similar agonist-induced activation. Washed platelets were incubated with control buffer, 50 ng/mL convulxin, or 0.5 U/mL thrombin and P-selectin expression was determined by flow cytometry (N = 4; ± S.E.M., NS by students T-test between WT and GRIP1^-/-^). B) PF4 release from stimulated WT and GRIP1^-/-^ platelets. Washed platelets were stimulated with thrombin or 2-meADP for 10 min. and PF4 release measured by ELISA (N = 8; ± S.E.M, N.S. by students—test). C) Washed WT and GRIP1^-/-^ platelets were thrombin-stimulated and ATP release was measured (N = 8; ± S.E.M, N.S. by students T-test). D) Washed WT and GRIP1^-/-^ platelets have similar GPIIb/IIIa activation. Platelets were stimulated with thrombin or 2-meADP for 10 min. Activated GPIIb/IIIa expression was measured by JON/A antibody binding. (N = 4; ± S.E.M, N.S. by students T-test). E) PRP from WT or GRIP1^-/-^ mice was incubated with either PE or APC labeled anti-CD9 antibody. PE and APC labeled PRP from mice of the same genotype was then mixed. Control buffer, thrombin or 2-meADP were added and incubated with orbital shaking at 37°C for 15 minutes. Double positive (APC and PE) platelet aggregates were quantified by flow cytometry. GRIP1^-/-^ platelets stimulated with low dose thrombin had a trend to fewer platelet aggregates compared to WT (N = 6–8). WT and GRIP1^-/-^ platelets stimulated with 2-meADP had similar aggregation.

After determining that platelet GRIP1^-/-^ mice have defective thrombosis, we next examined whether GRIP1 has a role in platelet activation. Washed WT and GRIP1^-/-^ platelets were isolated and stimulated with agonists, including convulxin (collagen receptor agonist) or thrombin, and platelet activation was determined by measuring surface P-selectin expression. WT and GRIP1^-/-^ platelets had similar P-selectin external membrane translocation in response to both agonists (Fig 3A). As further confirmation of similar platelet α-granule release, PF4 secretion from activated WT and GRIP1^-/-^ platelets was assessed. Thrombin and meADP induced similar PF4 secretion from WT and GRIP1^-/-^ platelets (Fig 3B). The dense granule constituent, ATP, was measured from thrombin-activated WT and GRIP1^-/-^ platelets and was unchanged between the two groups (Fig 3C). To examine GPIIb/IIIa activation, platelets were stimulated with thrombin and meADP and activated GPIIb/IIIa was assessed using JON/A antibody. WT and GRIP1^-/-^ platelets showed no difference in agonist-induced integrin activation (Fig 3D). These data indicate that GRIP1^-/-^ platelets have no changes in agonist activation.

We also assessed platelet aggregation using a flow cytometry-based method.^21^ Platelet rich plasma (PRP) from WT or GRIP1^-/-^ mice was incubated with either a PE or APC labeled anti-CD9 antibody. PE and APC-labeled PRP from mice of the same genotype was then mixed in an equal ratio. Platelets were activated with either thrombin or 2-meADP (P_2_Y_12_ agonist) and double labeled platelet aggregates measured by flow cytometry. GRIP1^-/-^ platelets had a trend to fewer platelet aggregates at low dose thrombin stimulation (Fig 3E). WT and GRIP1^-/-^ platelets had similar platelet aggregation at higher thrombin concentrations and with ADP stimulation (Fig 3E).

**Fig 4:**
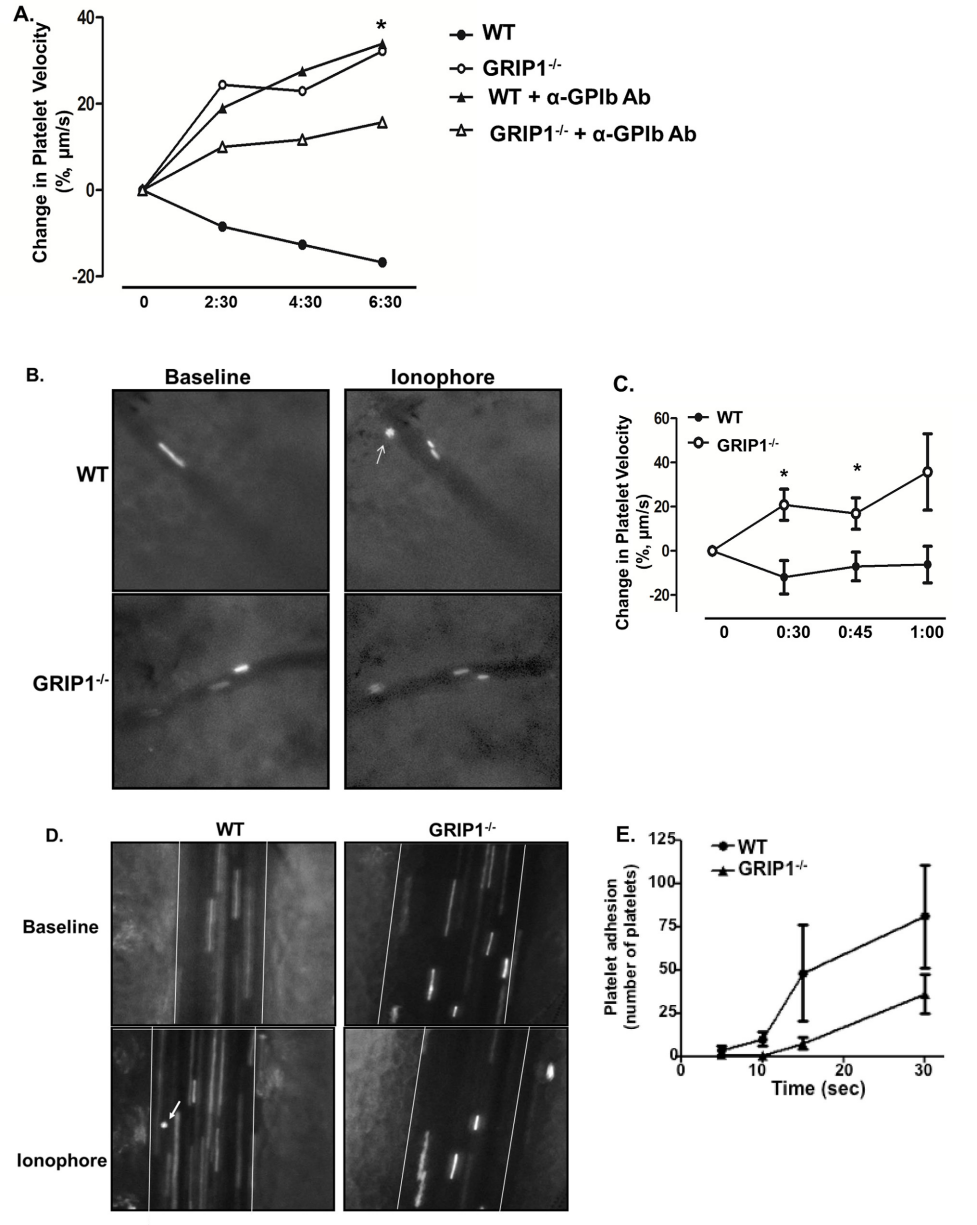
GRIP1^-/-^ platelets do not have decreased velocity in ionophore-stimulated blood vessels. A) GRIP1^-/-^ platelets do not have a decreased velocity in an ionophore-stimulated pinna vessel. WT murine pinna vessels were stimulated with A23187 and the change in fluorescent-labeled WT and GRIP1^-/-^ platelet velocity determined over time. As a control, platelets were treated with GPIbα blocking antibody (N = 4; *P < 0.05 by students T-test GRIP1^-/-^ vs WT). B) Representative images of WT and GRIP1^-/-^ platelets before and 6 minutes after vessel ionophore treatment (smaller, rounder platelets are rolling (arrow), magnification 20X). C) GRIP1^-/-^ platelets do not have decreased velocity in an ionophore-stimulated mesenteric vessel (N = 4 ± S.E.M; *P < 0.05 by students T-test GRIP1^-/-^ vs WT). D) Representative images of WT and GRIP1^-/-^ platelets before and after ionophore treatment in mesenteric arterioles (smaller, rounder platelets are rolling (arrow), magnification 20X). E) GRIP1^-/-^ platelets have a trend toward reduced vWF adhesion in vitro. Platelets in whole blood from WT and GRIP1^-/-^ mice were labeled with a fluorescent antibody. Blood was then pumped over a mouse vWF-coated MatTek chamber. Platelet adhesion was determined at multiple time points (N = 4–6 per time point, P < 0.1 at 15 sec).

Because platelet activation was normal in GRIP1^-/-^ platelets, but mice had a thrombosis defect, we next sought to determine whether GRIP1^-/-^ platelets have altered adhesion to stimulated endothelial cells using an in vivo platelet rolling assay. Platelets isolated from WT or platelet GRIP1^-/-^ mice were fluorescently labeled and injected into control WT mice so that only the fluorescent platelet genotype differed. Fluorescent platelets were visualized in the ear or externalized mesenteric venules and endothelial cell stimulation and vWF release was induced by surface application of calcium ionophore (A23187). Platelets were imaged both before and after ionophore application and the change in platelet velocity was measured. As expected, WT platelets exhibited decreased velocity post-ionophore in both the ear and mesenteric venules (Fig 4A and 4C). However, GRIP1^-/-^ platelets did not have a decrease in post-ionophore velocity (Fig 4A and 4C). To ensure the specificity of GPIb-mediated rolling, WT or GRIP1^-/-^ platelets were pre-treated with blocking antibody to the vWF adhesion site of GPIbα. With the blocking antibody, WT mice had similar rolling velocity as GRIP1^-/-^ platelets (Fig 4A). Representative images show rolling and adhered WT platelets after ionophore addition with no changes in the GRIP1^-/-^ platelets (Fig 4B and 4D). We recapitulated these adhesion data in vitro. Platelets were fluorescently labeled in whole blood and pumped through a mouse vWF coated flow chamber. WT platelets had a trend toward greater adhesion than GRIP1^-/-^ platelets at earlier time points (Fig 4E). WT and GRIP1^-/-^ platelets had no difference in adhesion when pumped through a collagen coated chamber (not shown). These data indicate that GRIP1^-/-^ platelets have reduced interactions with a stimulated endothelium in vivo.

### GRIP1 interacts with the GPIb complex

**Fig 5:**
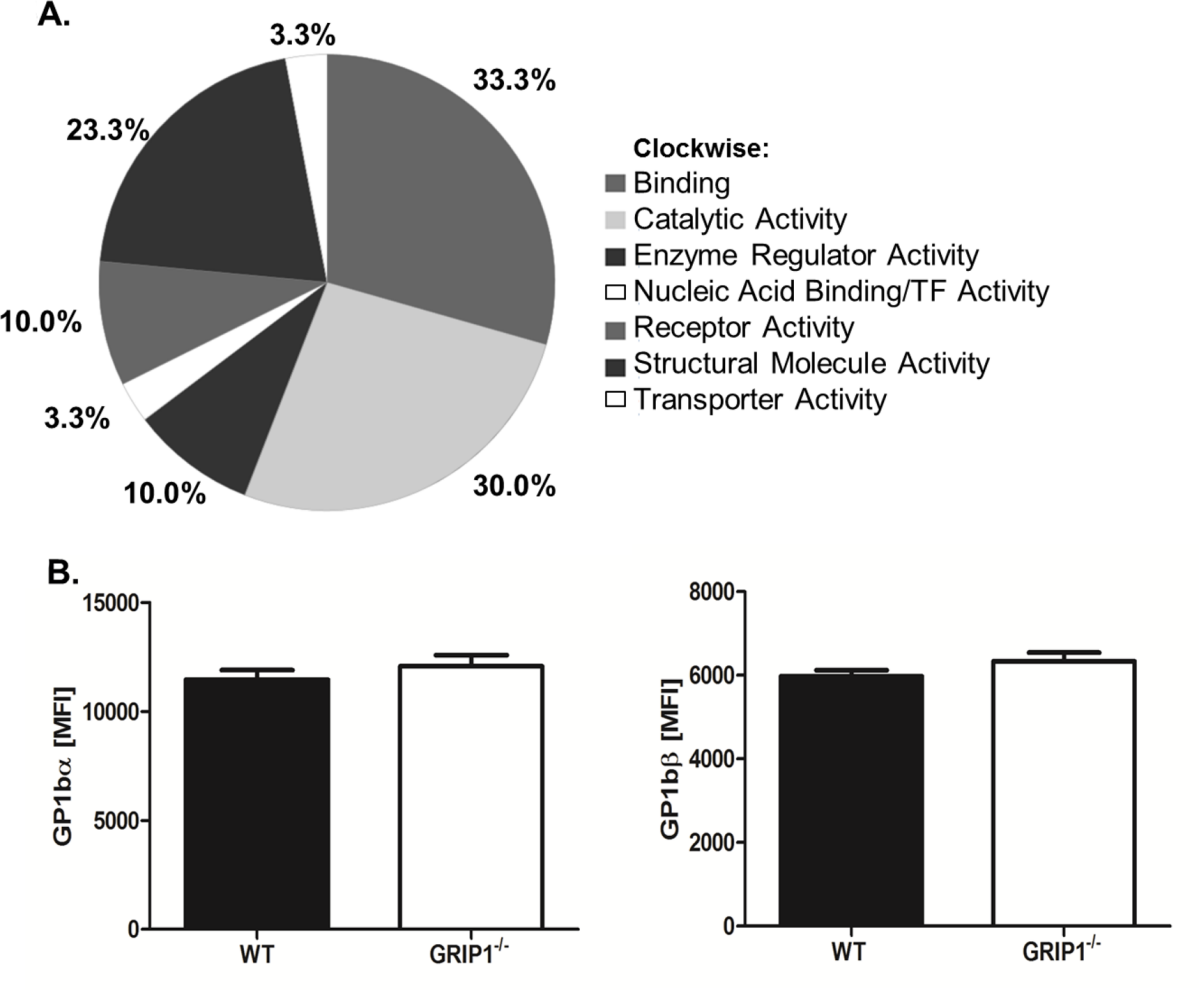
GRIP1 interacts with the GPIb-IX complex. A) GRIP1 protein interactions in platelets. GRIP1 was immunoprecipitated from WT and GRIP1^-/-^ (negative control) platelets and the precipitate analyzed by mass spectrometry. Proteins were assigned by Panther into GO functional groups. B) GPIbα and GPIbβ surface expression is similar in WT and GRIP1^-/-^ platelets. GPIbα and GPIbβ surface expression was determined by flow cytometry (N = 8–13, ± S.E.M; NS by students T-test).

To begin to define a mechanism for why GRIP1^-/-^ platelets have decreased interactions with a stimulated or damaged endothelium, we identified potential platelet GRIP1 interacting proteins. GRIP1 is a large multi-PDZ domain protein with many potential intracellular interactions. We immunoprecipitated GRIP1 from WT platelets, or as a negative control, GRIP1^-/-^ platelets, and performed mass spectrometry on the precipitate. Numerous GRIP1 interacting proteins were greatly enriched in the WT platelet immunoprecipitation compared to GRIP1^-/-^ platelets. GRIP1 interacting proteins were analyzed by GO analysis using Panther software [21] including multiple members of the GPIb-IX adhesion complex, including GPIbα, GPIbβ, and 14-3-3 (Fig 5A and Table 1). GRIP1 and 14-3-3 binding has been described in modified HEK293 cells [18] and neurons [19] and 14-3-3 is an important part of the GPIb-IX complex in platelets further indicating a potential role for GRIP1 in this complex.

**Table 1:** Proteins highly enriched in mass spectrometry analysis with known roles in platelet function (light gray indicates interact with GPIb complex, darker shading indicates other functional platelet protein interactions).

Protein Name	Function in Platelets	Interactions in Platelets
**GPIbα**	**Adhesion, integrin activation [20,25]**	**GPIbβ, GPV, GPIX, 14-3-3, PI3K, filamin-A, spectrins**
**GPIbβ**	**Adhesion, integrin activation [20]**	**GPIbα, GPV, GPIX, 14-3-3**
**14-3-3ζ**	**Adhesion, integrin activation [20]**	**GPIbα, GPIbβ, GPV, GPIX**
**Spectrin (multiple proteins)**	**Cytoskeletal (interacts with GPIb/actin binding complexes) [20]**	**Actin-binding proteins, GPIb-complex**
**Actin**	**Cytoskeletal [20]**	**Spectrins, GPIb-complex**
**Pleckstrin**	**Secretion, aggregation, actin polymerization [26]**	**PKCα (has PDZ domain)**
**Cyclophilin A**	**Secretion, activation, integrin activation [27]**	**SERCA2b**
**Integrin linked kinase**	**Activation, aggregation, secretion, thrombus formation [28]**	***β*–parvin**
**Zyxin**	**Adhesion, cytoskeletal interactions [29]**	**VASP proteins**

**Fig 6:**
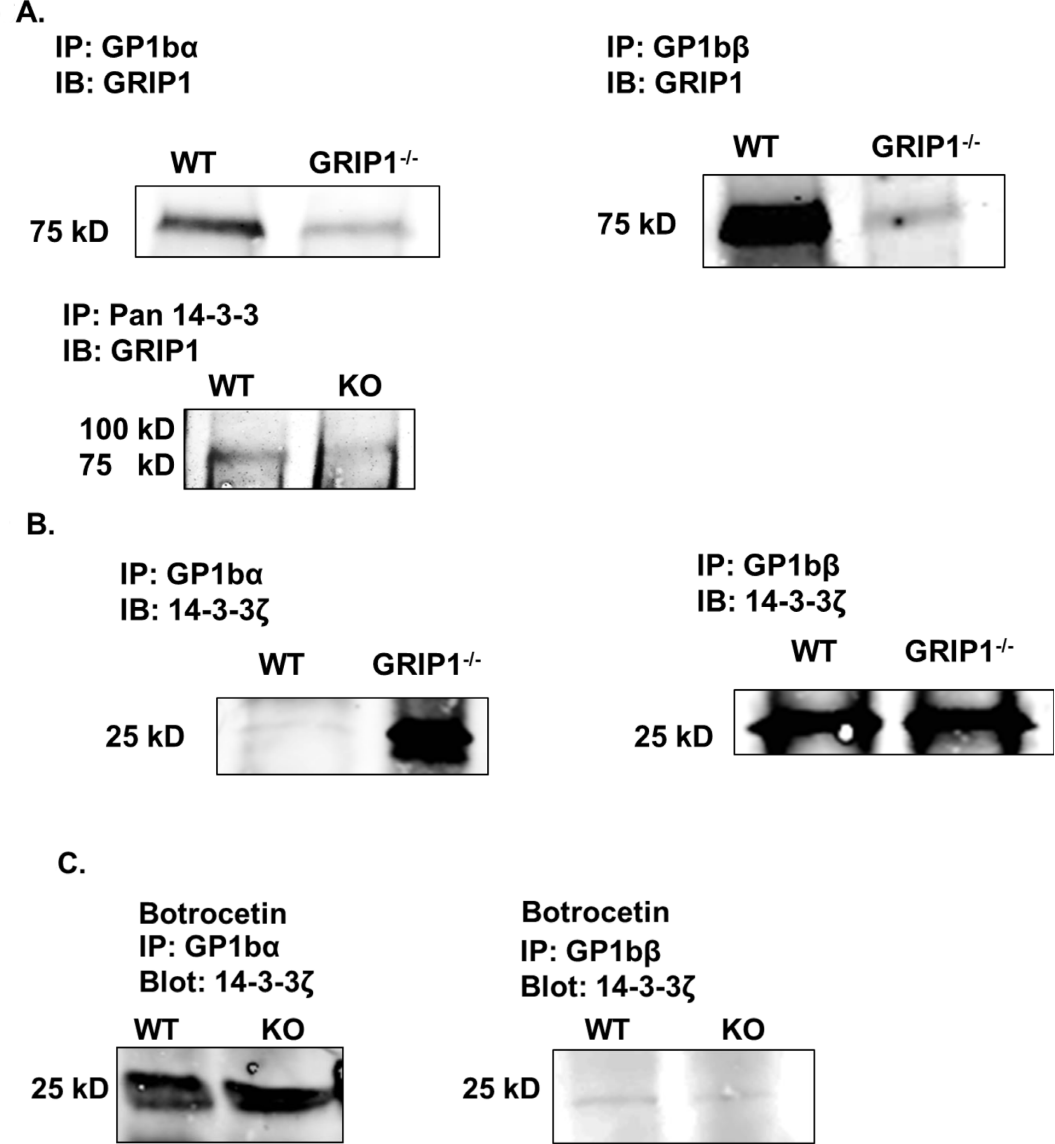
GPIb-IX adhesion complex interactions differ in WT and GRIP1^-/-^ platelets. A) GRIP1 interacts with GPIbα, GPIbβ, and 14-3-3. GPIbα, GPIbβ, or 14-3-3 were immoprecipitated and immunoblotted for GRIP1 (GRIP1^-/-^ platelets as negative control). B) GRIP1^-/-^ platelets have increased GPIbα and 14-3-3ζ interactions at rest, but no difference in GPIbβ interactions between WT and GRIP1^-/-^ (representative blots of multiple experiments). C) WT and GRIP1^-/-^ platelets have similar GPIbα-14-3-3ζ and GPIbβ-14-3-3ζ association post-stimulation with botrocetin and vWF (Representative blots of multiple experiments).

We next investigated whether deletion of GRIP1 affected surface expression of GPIbα and GPIbβ. There was no difference between WT and GRIP1^-/-^ platelet surface expression of GPIbα and GPIbβ (Fig 5B). We then confirmed protein interactions of GRIP1 with members of the GPIb-IX complex. GPIbα or GPIbβ was immunoprecipitated from WT and GRIP1^-/-^ platelets and the precipitate immunoblotted for GRIP1. As suggested by the mass spectrometry data, GRIP1 interacted with GPIbα and GPIbβ in WT platelets, but not in GRIP1^-/-^ platelets (Fig 6A). We also found that GRIP1 interacted with 14-3-3 in platelets (Fig 6A).

To determine if adhesion complex interactions are altered in the absence of GRIP1, we used WT and GRIP1^-/-^ platelets to immunoprecipitate GPIbα and GPIbβ and determined whether 14-3-3 interacted. While GPIbβ and 14-3-3 interacted strongly in WT and GRIP1^-/-^ platelets, GPIbα only strongly interacted with 14-3-3 in GRIP1^-/-^ platelets (Fig 6B). We incubated WT and GRIP1^-/-^ platelets in vitro with botrocetin and vWF to determine if there were binding partner alterations after botrocetin-induced vWF binding and GPIb complex activation. In contrast to basal conditions where WT platelets showed a weak interaction between 14-3-3ζ and GPIbα, botrocetin-activated WT and GRIP1^-/-^ platelets had similar associations between 14-3-3ζ and GPIbα (Fig 6C). 14-3-3ζ and GPIbβ interactions were similarly reduced in both WT and GRIP1^-/-^ platelets after botrocetin-vWF incubation (Fig 6C). These data suggest that GRIP1 limits the association of 14-3-3ζ and GPIbα at rest and may be important for GPIb-IX complex function and signaling.

Taken together, these findings indicate that GRIP1 interacts with the GPIb-IX complex and that GRIP1 is needed for efficient thrombus formation in vivo.

## Discussion

While the mechanism of GPIb-IX complex adhesion to vWF is well-studied, less is known about intracellular interactions of the GPIb-IX complex. Other groups have found that the GPIb-IX complex binds to 14-3-3, filamin A, calmodulin, and phosphoinositol-3 kinase [20]. We have found that GRIP1 is present in mouse and human platelets and that GRIP1 binds to the GPIb-IX complex, either directly or involving an interaction with 14-3-3. Deletion of GRIP1 from platelets causes a significant decrease in platelet interactions with a stimulated endothelial cell layer and delayed thrombus formation. GRIP1^-/-^ platelets also have changes in GPIb-IX complex molecular associations (including with 14-3-3) during resting conditions. Understanding GRIP1 protein interactions in platelets may add knowledge to platelet adhesion and signaling.

Defects in GRIP1^-/-^ platelet function are similar to those noted in other mouse models in which GPIbα [30, 31] or vWF [32] are mutated. Similar to mice lacking GRIP1 in platelets, mice modified to have the extracellular domain of GPIbα replaced with the extracellular interleukin-4 receptor (IL4Rα/GPIb transgene) had normal platelet numbers and morphology, but a significant decrease in thrombus formation and platelet incorporation into the growing thrombus [30]. Additionally, GPIbα deficiency produces a more profound phenotype than vWF deletion alone [32,33] indicating other important functions for the GPIb-IX complex besides vWF adhesion. Complete deletion of GPIbα increases bleeding times similar to those seen with GRIP1^-/-^ mice [31]. However, we did not see changes in platelet size, nor alterations in GPIbα surface expression in platelet GRIP1^-/-^ mice. This indicates that while surface expression of GPIbα is intact in GRIP1^-/-^ platelets, GPIbα intracellular interactions and signaling may be altered leading to defects in extracellular GPIb complex function.

Both GPIbα and GPIbβ are reported to have 14-3-3ζ binding sites [20]. Our data indicates that GPIbα does not strongly bind 14-3-3ζ under resting conditions, but in the absence of GRIP1, GPIbα has increased interaction with 14-3-3ζ. The GPIbα and 14-3-3 basal interactions we noted are supported by a recent study that found similar interactions before and after shear stress [34]. Others have suggested more robust GPIbα and 14-3-3ζ interactions in resting platelets [35]. These differences in data may be due to experimental conditions or antibodies used, but all point to the complexity of GPIbα interactions that may be regulated by phosphorylation status or yet to be defined members of the GPIb-IX complex [20]. GPIb-IX activation in response to botrocetin and vWF resulted in similar GPIbα and GPIbβ interactions with 14-3-3ζ in both WT and GRIP1^-/-^ platelets. This suggests that in the resting state, 14-3-3ζ is bound primarily to GPIbβ, and after activation, 14-3-3ζ is predominantly bound to GPIbα. Our data indicates that GRIP1 limits GPIbα and 14-3-3 interactions in basal conditions. It has also recently been demonstrated that GPIb-IX cooperates with protease activated receptor (PAR) signaling to promote platelet responses to low thrombin concentrations [36]. Our data indicated that GRIP1^-/-^ platelets may have less aggregation with low dose thrombin. This may be confirmation that GRIP1 participates in the GPIb-IX complex response to low dose thrombin signaling.

Additional studies are needed to fully elucidate GRIP1 mediated signaling events in the GPIb-IX complex in both resting and stimulated states. Our study may also have larger implications beyond platelet adhesion alone as the GPIb-IX complex has known functions outside of platelet adhesion such as bacterial recognition [37–38] and platelet clearance [39, 40]. We have found that GRIP1 has important roles in platelet function through potential changes in intracellular interactions of the GPIb-IX complex.

## Abbreviations

GRIP1: glutamate receptor interacting protein 1
vWF: von Willebrand factor
PDZ: post-synaptic density 95, discs large tumor suppressor, and zonula occludens 1
